# Ligubenzocycloheptanone A, a Novel Tricyclic Butenolide with a 6/7/5 Skeleton from *Ligusticum chuanxiong*

**DOI:** 10.1038/srep28783

**Published:** 2016-07-27

**Authors:** Bing Han, Xu Zhang, Zi-Ming Feng, Jian-Shuang Jiang, Li Li, Ya-Nan Yang, Pei-Cheng Zhang

**Affiliations:** 1 State Key Laboratory of Bioactive Substance and Function of Natural Medicines, Institute of Materia Medica, Chinese Academy of Medical Sciences and Peking Union Medical College, Beijing 100050, P. R. China

## Abstract

Ligubenzocycloheptanone A (**1**), a novel tricyclic butenolide with a 6/7/5-membered ring skeleton, was isolated from the rhizome of *Ligusticum chuanxiong*. Its unusual structure was determined using UV, IR, HRESIMS, 1D and 2D NMR data, X-ray diffraction crystallography and by the comparison of experimental and calculated electronic circular dichroism (ECD) spectra. **1** possessed a benzocycloheptanone core featuring butyrolactone, which is rarely observed in nature. A possible biosynthetic pathway was proposed. Ligubenzocycloheptanone A showed strong radical scavenging activity with an IC_50_ value of 2.3 *μ*M.

*Ligusticum chuanxiong* Hort (Umbelliferae) is mainly distributed in Sichuan province in China. Its dried rhizome is used in traditional Chinese medicine for the treatment of headaches, rheumatic arthralgia, menstrual disorders, swelling pain due to traumatic injury, and cardiovascular and cerebrovascular diseases^1^,^2^,^3^. Phytochemical investigations of this plant have reported the presence of phenolic acids^4^, alkaloids^5^, and phthalides^6^,^7^,^8^. Among these compounds, phthalides are usually considered to be the active constituents of *Ligusticum chuanxiong* with the effects of inhibiting vasoconstriction, antiproliferation, antioxidation, and antiinflammation^9^,^10^,^11^,^12^. In our search for additional active constituents from this plant, a novel tricyclic butenolide with an uncommon 6/7/5-membered ring skeleton, named ligubenzocycloheptanone A (Fig. 1), was isolated from the rhizome of *Ligusticum chuanxiong*. Its structure was determined using UV, IR, HRESIMS, 1D and 2D NMR data, X-ray diffraction crystallography, and by comparing experimental and calculated electronic circular dichroism (ECD) spectra. Ligubenzocycloheptanone A may be derived biosynthetically from a phenyl acrylic acid and a hexose diacid. This may be useful for the exploration of a new synthetic approach to benzocycloheptanone derivatives. Ligubenzocycloheptanone A showed strong radical scavenging activity with an IC_50_ value of 2.3 *μ*M.

## Results

Compound **1** was isolated as a light yellow amorphous powder. Its molecular formula, C_15_H_12_O_8_, was established using HRESIMS with *m/z* 321.0593 [M+H]^+^ (calcd 321.0605), which requires 10 degrees of unsaturation. Its IR spectrum exhibited absorptions at 3374.4 cm^−1^ (hydroxy), 1741.4 and 1667.2 cm^−1^ (carbonyl), and 1587.9 cm^−1^ (aromatic rings).

The ^1^H NMR spectrum of **1** displayed two *ortho*-coupled aromatic protons at *δ*_H_ 7.06 (1H, d, *J* = 8.0 Hz, H-5) and 7.13 (1H, d, *J* = 8.0 Hz, H-6) and one olefinic proton with an allylic coupling at *δ*_H_ 7.29 (1H, d, *J* = 3.0 Hz, H-7). Additionally, a characteristic downfield proton signal at *δ*_H_ 13.31 (1H, s, 3-OH) suggested the presence of an internal hydrogen bond. Furthermore, two oxymethines at *δ*_H_ 4.59 (1H, dd, *J* = 2.0, 6.5 Hz, H-4′) and 4.34 (1H, d, *J* = 2.0 Hz, H-5′), one methine at *δ*_H_ 3.41 (1H, m, H-3′), and one methylene at *δ*_H_ 2.93 (1H, d, *J* = 13.0 Hz, H-2′a) and 3.37 (1H, t, *J* = 13.0 Hz, H-2′b) were observed in the ^1^H NMR spectrum.

The ^13^C NMR (Table 1) and HSQC spectra revealed a total of 15 carbon signals: one methylene, six methines, and eight quaternary carbons. Eight sp^2^ carbons at *δ*_C_ 153.7 (C-3), 149.3 (C-4), 135.7 (C-7), 128.9 (C-8), 127.8 (C-6), 125.2 (C-1), 119.5 (C-5), and 118.5 (C-2) could be attributed to six aromatic carbons and two olefinic carbons, suggesting the presence of a tetrasubstituted benzene ring and an olefinic moiety. The substitution pattern of the benzene ring was determined mainly by HMBC correlations (Fig. 2). In the HMBC spectrum, one aromatic proton at *δ*_H_ 7.06 (1H, d, *J* = 8.0 Hz, H-5) showed strong HMBC correlations with two carbons at *δ*_C_ 125.2 (C-1) and 153.7 (C-3), and the other one at *δ*_H_ 7.13 (1H, d, *J* = 8.0 Hz, H-6) showed correlations with three carbons at *δ*_C_ 118.5 (C-2), 149.3 (C-4), and 135.7 (C-7). In combination with the key correlations from the olefinic proton at *δ*_H_ 7.29 (1H, d, *J* = 3.0 Hz, H-7) to C-1, C-2, C-6, C-8, and C-9, a 3,4-dihydroxycinnamoyl moiety in **1** was clearly established.

In the ^1^H-^1^H COSY experiment (Fig. 2), the correlations of H-3′ with H-2′ and H-4′, and H-4′ with H-3′ and H-5′, suggested the presence of -CH_2_^_^CH-CH-CH- in **1**. Combined with the HMBC correlations from H-2′ and H-3′ to C-1′ at *δ*_C_ 204.8 and H-4′ and H-5′ to C-6′ at *δ*_C_ 172.7, these results suggested the existence of a hexaric acid moiety. Furthermore, in the HMBC experiment, the correlation of H-2′a with C-2 confirmed that the hexaric acid moiety was connected to C-2 of the tetrasubstituted benzene ring by a C-C bond, while the HMBC correlations of H-3′ with C-7 and C-8 and H-2′ with C-8 suggested that C-3′ was connected to C-8. A benzocycloheptanone skeleton was formed. The absence of other sp or sp^2^ carbon signals and the remaining 1 degree of unsaturation implied that **1** contained a lactone ring, which was further confirmed by the HMBC correlation of H-4′ with C-9.

The relative configuration of **1** was deduced through the ROESY experiment. In the ROESY experiment (Fig. 3), the key correlations of H-4′ with H-2′a and H-2′b indicated the *trans* configuration of H-3′ and H-4′.

The absolute configuration of C-3′, C-4′, and C-5′ in **1** was established by comparing the experimental CD spectrum and the calculated ECD data (Supporting Information). Considering the *trans* configuration of H-3′ and H-4′, **1** has only two pairs of enantiomers (**1a**: 3′*S*,4′*S*,5′*R* and **1b**: 3′*R*,4′*R*,5′*S*; **1c**: 3′*S*,4′*S*,5′*S* and **1d**: 3′*R*,4′*R*,5′*R*). A systematic conformational analysis was performed for **1a** and **1c** using a MMFF94 molecular mechanics force field calculation. The optimized conformations of **1a** and **1c** were obtained using the time-dependent density functional theory (TD-DFT) method at the B3LYP/6-31G(d) level. The overall calculated ECD spectra of **1a**, **1b**, **1c**, and **1d** were generated by Boltzmann weighting of their lowest energy conformers. The overall pattern of the calculated ECD spectrum of **1b** attributable to the 3′*R*,4′*R*,5′*S*-isomer was consistent with the experimental data for **1** throughout the entire range of wavelengths under investigation (Fig. 4). Accordingly, the dihedral angles of H2′aC2′C3′H3′ (−71°), H2′bC2′C3′H3′ (170°), H3′C3′C4′H4′ (145°), and H4′C4′C5′H5′ (−59°) in the optimized conformation (Fig. 3) favored the coupling constants of H-2′a (d, *J* = 13.0 Hz), H-2′b (t, *J* = 13.0 Hz), H-4′ (dd, *J* = 2.0, 6.5 Hz), and H-5′ (d, *J* = 2.0 Hz).

**Figure 1:**
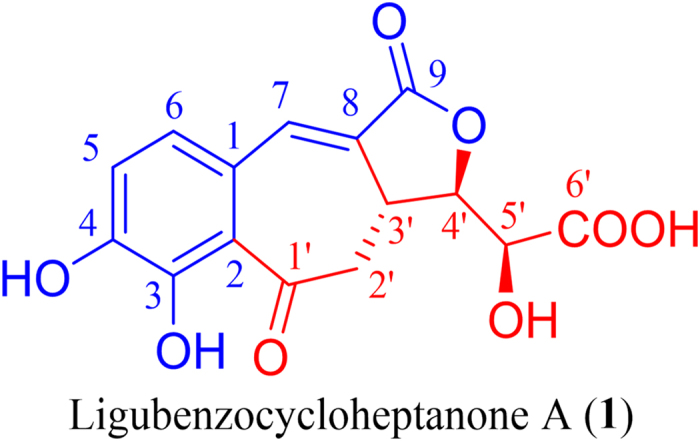
Structure of ligubenzocycloheptanone A.

**Figure 2:**
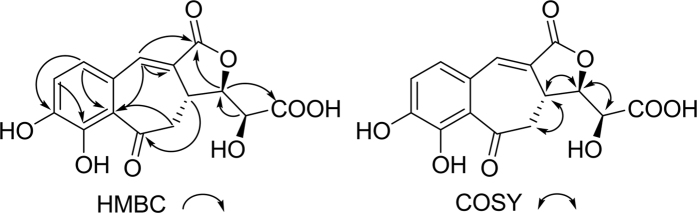
Key HMBC and COSY correlations of 1.

**Figure 3:**
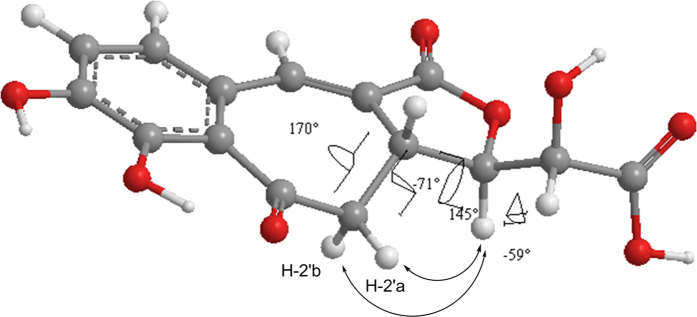
The optimized conformation and the key ROESY correlations of 1.

**Figure 4:**
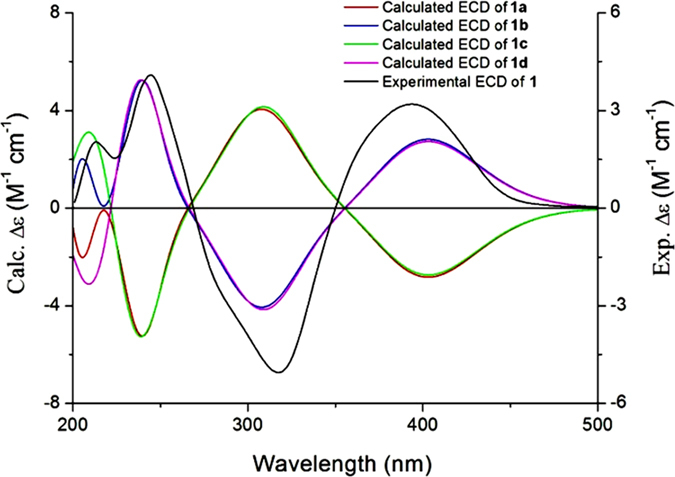
Experimental ECD spectrum of **1** and calculated ECD of **1a**, **1b**, **1c** and **1d** in MeOH.

**Figure 5:**
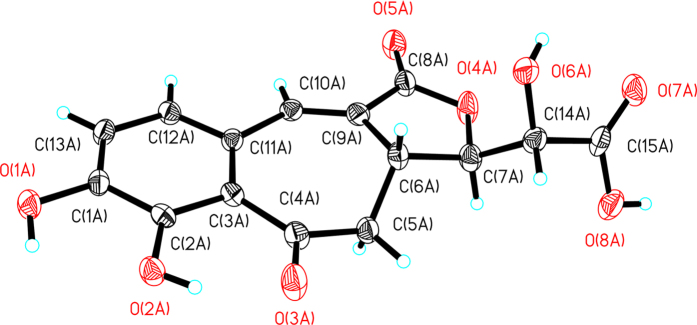
X-ray crystal structure of 1.

Fortunately, the crystal of **1** was obtained in the solvent of MeOH:H_2_O (9:1). Analysis of single crystal X-ray diffraction unambiguous proved the 3′*R*,4′*R*,5′*S* configurations for **1** (Fig. 5). Based on the above evidence, the structure of **1** was determined to be as shown in Fig. 1 and was named ligubenzocycloheptanone A.

## Discussion

**Figure 6:**
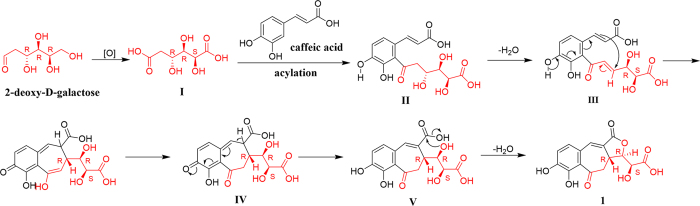
Plausible biogenetic pathway of 1.

Recently, benzotropone and benzocycloheptanone derivatives have attracted substantial attention^13^,^14^,^15^,^16^,^17^,^18^,^19^. Obviously, **1** represents a novel tricyclic butenolide with a benzocycloheptanone core. Its most intriguing feature from a biosynthetic pathway perspective is that **1** may be derived from a phenyl acrylic acid and a hexose diacid. 2-Deoxy-D-galactose and caffeic acid are considered to be precursors in a plausible biogenetic pathway of **1** (Fig. 6). One molecule of I formed from 2-deoxy-D-galactose by oxidation reacted with caffeic acid in a manner similar to a Friedel-Crafts acylation to give II, followed by dehydration, intramolecular nucleophilic addition, and hydride shift to give V. Finally, a five-membered lactone ring was formed by intramolecular nucleophilic substitution. The key process was the formation of the cycloheptanone moiety by the addition of the double bond in the caffeic acid unit to an *α*,*β*-unsaturated ketone. This information may be of considerable interest for the development of a new and efficient synthetic approach to benzocycloheptanone derivatives. In the *in vitro* bioactivity assays^24^ (Supporting Information), **1** showed strong radical scavenging activity with an IC_50_ value of 2.3 *μ*M, using ascorbic acid as a positive control.

## Methods

### General experimental procedures

The optical rotations were measured on a Jasco P-2000 polarimeter. IR spectra were recorded on an IMPACT 400 (KBr) spectrometer. ^1^H NMR (500 MHz), ^13^C NMR (125 MHz), and 2D-NMR spectra were run on INOVA 500 spectrometers. HRESIMS were performed on Agilent 6520 LC-Q-TOF mass spectrometer (Agilent Technologies, Waldbronn, Germany). Column chromatography was performed with macroporous resin (Diaion HP-20, Mitsubishi Chemical Corp., Tokyo, Japan), Rp-18 (50 *μ*m, YMC, Kyoto, Japan), Sephadex LH-20 (Pharmacia Fine Chemicals, Uppsala, Sweden). Preparative HPLC was carried out on a Shimadzu LC-6AD instrument with an SPD-20A detector, using a YMC-Pack ODS-A column (250 mm × 20 mm, 5 *μ*m). HPLC-diode array detection (DAD) analysis was performed on an Agilent 1200 series system with an Apollo C18 column (250 mm × 4.6 mm, 5 *μ*m, Alltech Corp., Kentucky, USA).

### Plant material

The rhizomes of *Ligusticum chuanxiong* Hort. were collected from Pengzhou Town, Sichuan Province in People’s Republic of China, in Jun 2013. The plant material was identified by Ma Lin (Institute of Materia Medica, Peking Union Medical College and Chinese Academy of Medical Sciences, Beijing 100050, P R. China). A voucher specimen (ID-S-2594) was deposited at the Institute of Materia Medica, Peking Union Medical College and Chinese Academy of Medical Sciences, Beijing 100050, People’s Republic of China.

### Extraction and isolation

Air-dried powder rhizome of *Ligusticum chuanxiong* Hort. (100.0 kg) were exhaustively extracted with 80% EtOH under refluxed conditions. After the solvent was evaporated under reduced pressure, the residue (23.1 kg) was suspended in water (50 L) and partitioned successively with EtOAc and n-BuOH. The n-BuOH-soluble portion (1300 g) was chromatographed on macroporous adsorption resins (HP-20) column, eluting with H_2_O, 15% ethanol, 30% ethanol, 50% ethanol, and 95% ethanol to give fractions A–E, respectively. Fr. C (103.0 g) was chromatographed over reversed phase silica gel column eluting with H_2_O-MeOH (from 100:0 to 0:100) to give 16 fractions (Fr. C-1–C-16). Fr. C-14 was further purified by Sephadex LH-20 column chromatography and preparative HPLC to yield compound **1** (19 mg).

ligubenzocycloheptanone A (**1**): 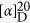 44.5^°^ (*c* 0.10, MeOH); IR *ν*_max_ 3374, 2955, 2708, 1741, 1667, 1618, 1588, 1435, 1376, 1302, 1198, 1021 cm^−1^; UV (MeOH) *λ*_max_ (log *ε*): 200 (4.04), 247 (4.18), 307 (3.81), 398 (3.82) nm; For ^1^H and ^13^C NMR spectroscopic data, see Table 1; HRESIMS *m/z*: 321.0593 (calcd for C_15_H_12_O_8_, 321.0605).

## Additional Information

**How to cite this article**: Han, B. *et al.* Ligubenzocycloheptanone A, a Novel Tricyclic Butenolide with a 6/7/5 Skeleton from *Ligusticum chuanxiong. Sci. Rep.* **6**, 28783; doi: 10.1038/srep28783 (2016).

## Supplementary Material

Supplementary Information

## Figures and Tables

**Table 1:** NMR data of compound 1 at 500 MHz in DMSO-*d*_6_.

Position	*δ*_H_	*δ*_C_	HMBC
1		125.2	
2		118.5	
3		153.7	
4		149.3	
5	7.06, d (8.0)	119.5	C-1, 3, 4, 6
6	7.13, d (8.0)	127.8	C-1, 2, 4, 5, 7
7	7.29, d (3.0)	135.7	C-1, 2, 6, 8, 9, 3′
8		128.9	
9		169.4	
1′		204.8	
2′a 2′b	2.93, d (13.0) 3.37, t (13.0)	44.9	C-2, 8, 1′, 3′, 4′ C-8, 1′, 3′, 4′
3′	3.41, m	33.7	C-7, 8, 9, 1′, 2′, 4′, 5′
4′	4.59, dd (2.0, 6.5)	81.9	C-9, 2′, 3′, 5′, 6′
5′	4.34, d (2.0)	68.6	C-3′, 4′, 6′
6′		172.7	
