# Supplementary material for: Ligubenzocycloheptanone A, a Novel Tricyclic Butenolide with a 6/7/5 Skeleton from Ligusticum chuanxiong
Source: Sci Rep. 2016 Jul 27;6:28783. doi: 10.1038/srep28783 (PMC4962078; doi:10.1038/srep28783)
Supplement: Supplementary Information [file srep28783-s1.pdf]

Ligubenzocycloheptanone A, a Novel Tricyclic Butenolide with a 6/7/5 Skeleton from *Ligusticum chuanxiong*

Bing Han, Xu Zhang, Zi-Ming Feng, Jian-Shuang Jiang, Li Li, Ya-Nan Yang*, Pei-Cheng Zhang*

State Key Laboratory of Bioactive Substance and Function of Natural Medicines, Institute of Materia Medica, Chinese Academy of Medical Sciences and Peking Union Medical College, Beijing 100050, P. R. China

Corresponding author: pczhang@imm.ac.cn, yyn@imm.ac.cn

Supporting information

The List of Contents

No	Content	Page
1	**Experimental section**	S3
2	**Figure S1.** Two pairs of enantiomers (**1a**, **1b**, **1c**, **1d**) of **1**.	S4
3	**Table S1**. Conformational Analysis of **1a**.	S4
4	**Table S2**. Conformational Analysis of **1c**.	S5
5	**Figure S2.** The experiment ECD spectrum of **1** and the calculated ECD spectrum of **1a** and **1b**.	S6
6	**Figure S3.** The experiment UV spectrum of **1** and the calculated UV spectrum of **1a**/**1b**.	S6
7	**Figure S4.** The experiment ECD spectrum of **1** and the calculated ECD spectrum of **1c** and **1d**.	S7
8	**Figure S5.** The experiment UV spectrum of **1** and the calculated UV spectrum of **1c**/**1d**.	S7
9	**Figure S6.** X-ray crystal structure of **1**.	S8
10	**Figure S7.** The IR spectrum of ligubenzocycloheptanone A (**1**)	S9
11	**Figure S8.** The HR-ESI-MS of ligubenzocycloheptanone A (**1**)	S9
12	**Figure S9.** The ^{1}H NMR spectrum of ligubenzocycloheptanone A (**1**) in DMSO-d_6	S10
13	**Figure S10.** The ^{13}C NMR spectrum of ligubenzocycloheptanone A (**1**) in DMSO-d_6	S10
14	**Figure S11.** The HSQC spectrum of ligubenzocycloheptanone A (**1**) in DMSO-d_6	S11
15	**Figure S12.** The HMBC spectrum of ligubenzocycloheptanone A (**1**) in DMSO-d_6	S11
16	**Figure S13.** The COSY spectrum of ligubenzocycloheptanone A (**1**) in DMSO-d_6	S12
17	**Figure S14.** The NOESY spectrum of ligubenzocycloheptanone A (**1**) in DMSO-d_6	S12

Experimental section

DPPH Radical Scavenging Assay. 100 μL methanolic solution of **1** (1×10^{-5} mol/L) were added to 150 μL solution of DPPH (6.1×10^{-5} mol/L) in methanol. The mixture was kept in the dark for 60 min at room temperature and then the decrease in absorption was measured. Absorbance was measured at 520 nm using an Ultramark microplate reader. Absorption of blank sample containing the same amount of methanol and DPPH solution was prepared and measured. The experiment was carried out in triplicate. Radical scavenging activity was calculated by the following formula:

$$\text{Inhibition (\%)} = [(OD_{(control)} – OD_{(sample)})/OD_{(control)}] \times 100$$

The antioxidant activity of **1** was expressed as IC_{50}. The IC_{50} value is defied as the concentration (mol/L) of **1** required for inhibiting the formation of DPPH radical by 50%. Sodium ascorbate was used as a positive control.

Determination of the absolute configuration of ligubenzocycloheptanone A (1) by measurement of the electronic circular dichroism (ECD) spectrum and by comparison with calculated ECD data. There were three chiral carbon atoms in ligubenzocycloheptanone A (**1**). The *erythro* comfiguration for C-3' and C-4' was deduced through the ^{1}H NMR coupling constants, the ROESY experiment, and systematic conformational analysis. Therefore **1** has a total of two pairs of enantiomers (**1a** and **1b**; **1c** and **1d**) (Figure S1). Conformational analysis of two diastereoisomers (**1a** and **1c**) of **1** was performed by using the MMFF94 molecular mechanics force field. The molecule of **1a** showed 10 lowest energy conformers (Table S1). The conformers were further optimized at the B3LYP/6-31g(d) level, 10 lowest electronic transitions were calculated. ECD spectra of different conformers were simulated using a Gaussian function with a half-bandwidth of 0.4eV. The overall theoretical ECD spectra were obtained according to the Boltzmann weighting of each conformers. The theoretically calculated ECD and UV spectra of **1b** (Figure S2) were in good agreement with the experimental ECD and UV spectra of **1** (Figure S3). Using the same method as that of **1a**, the conformational analysis of **1c** were completed (Table S2). The theoretically calculated ECD spectra of **1c** and **1d** were not in good agreement with the experimental ECD spectra of **1** (Figure S4). This allowed the assignment of the 3'*R*,4'*R*,5'*S* configurations for **1**.

1a	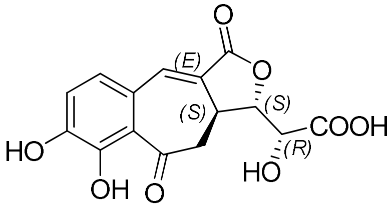	1c	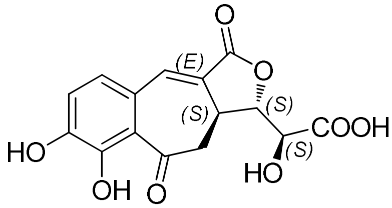
1b	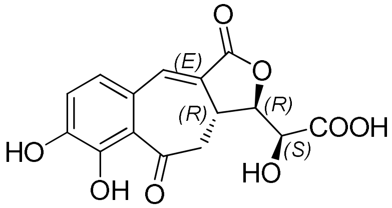	1d	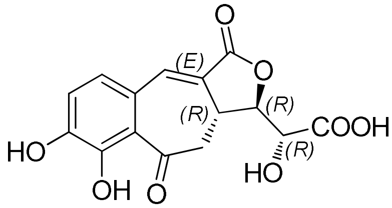

Figure S1. Two pairs of enantiomers (**1a, 1b, 1c, 1d**) of **1**.

Table S1 Conformational Analysis of **1a**

no.	conformer	population	no.	conformer	population
1a-C1	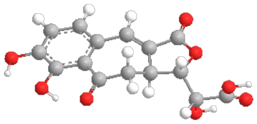	42.47%	1a-C6	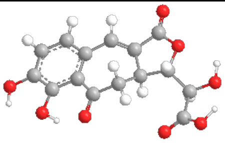	1.72%
1a-C2	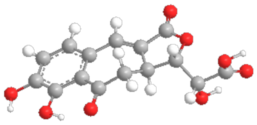	37.20%	1a-C7	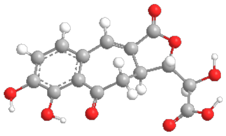	1.62%
1a-C3	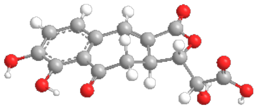	7.22%	1a-C8	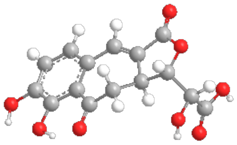	1.14%
1a-C4	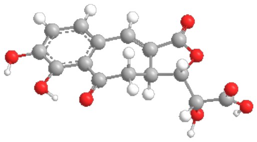	6.55%	1a-C9	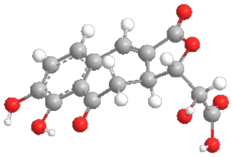	0.18%
1a-C5	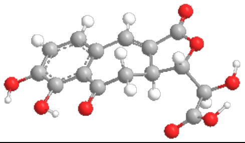	1.78%	1a-C10	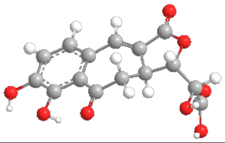	0.13%

Table S2. Conformational Analysis of **1c**

no.	conformer	population	no.	conformer	population
1c-C1	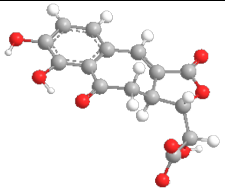	24.91%	1c-C6	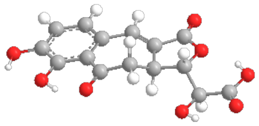	7.71%
1c-C2	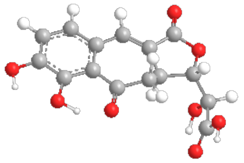	23.08%	1c-C7	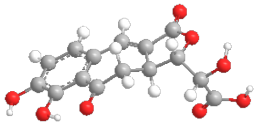	1.59%
1c-C3	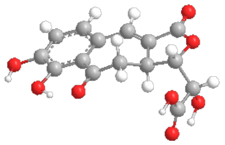	18.74%	1c-C8	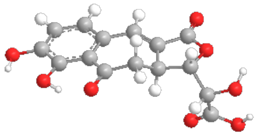	1.29%
1c-C4	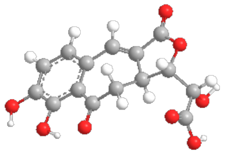	13.93%	1c-C9	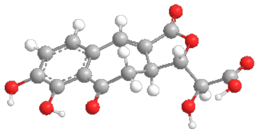	0.53%
1c-C5	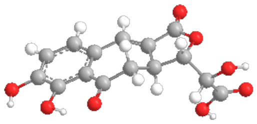	7.98%	1c-C10	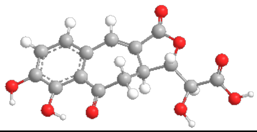	0.24%

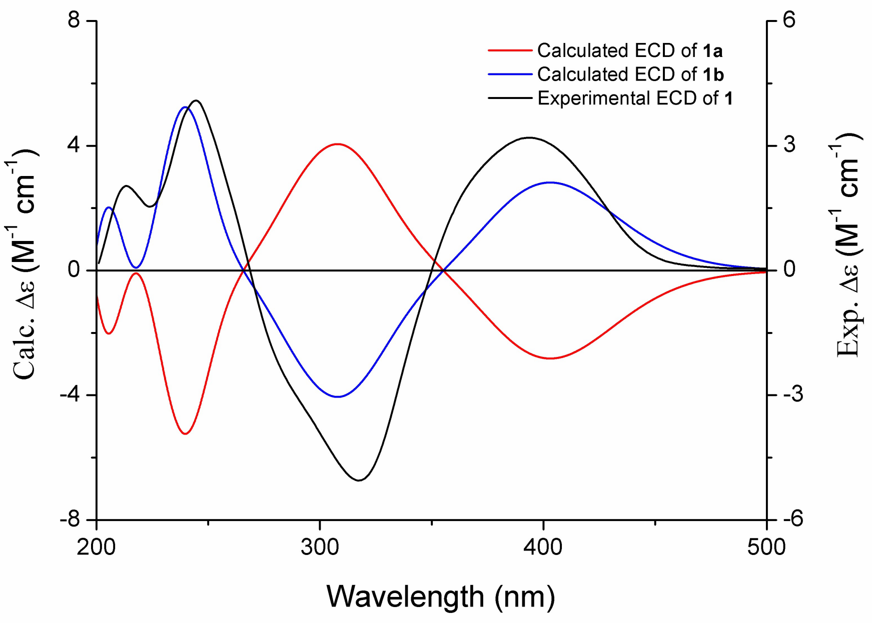

Figure S2. The experiment ECD spectrum of **1** and the calculated ECD spectrum of **1a** and **1b**.

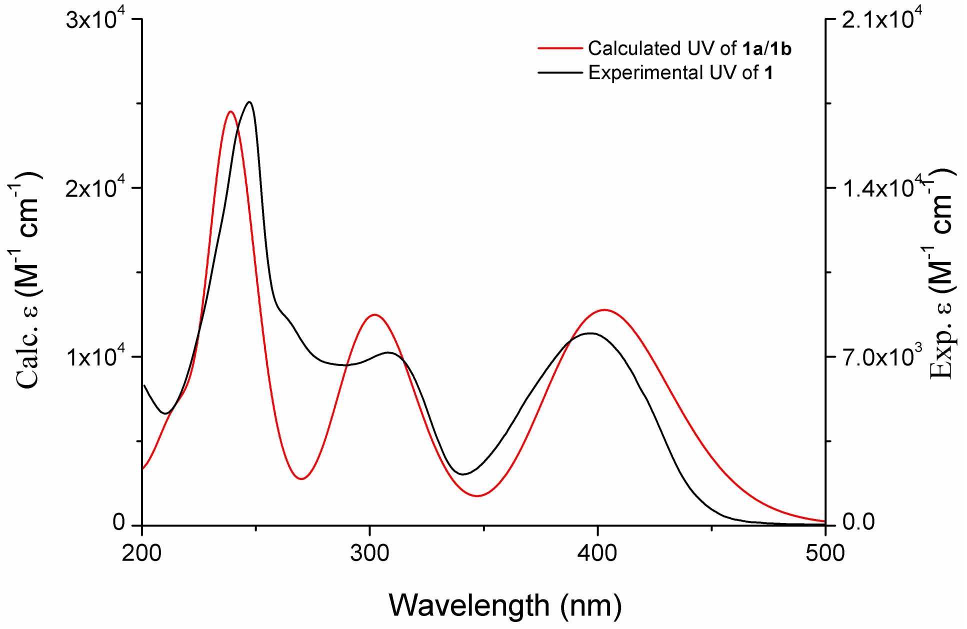

Figure S3. The experiment UV spectrum of **1** and the calculated UV spectrum of **1a**/**1b**.

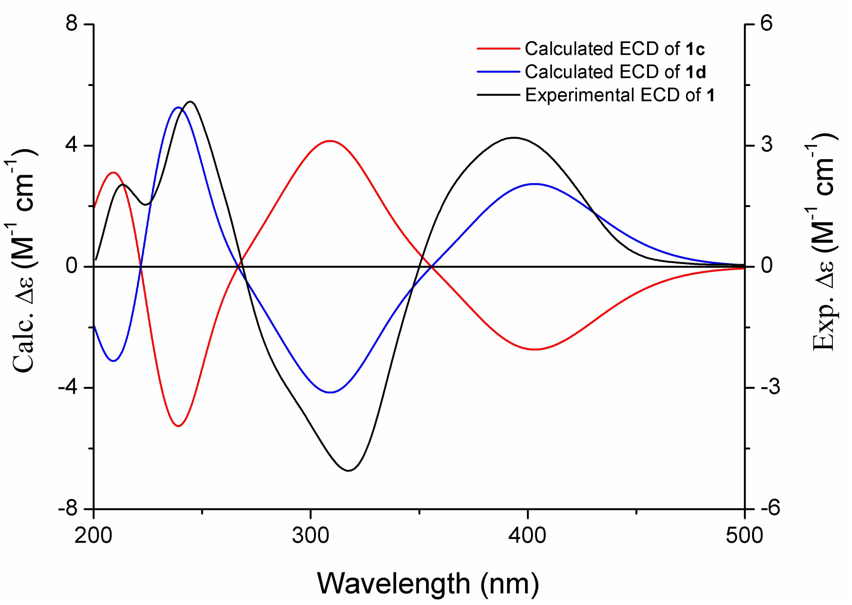

Figure S4. The experiment ECD spectrum of **1** and the calculated ECD spectrum of **1c** and **1d**.

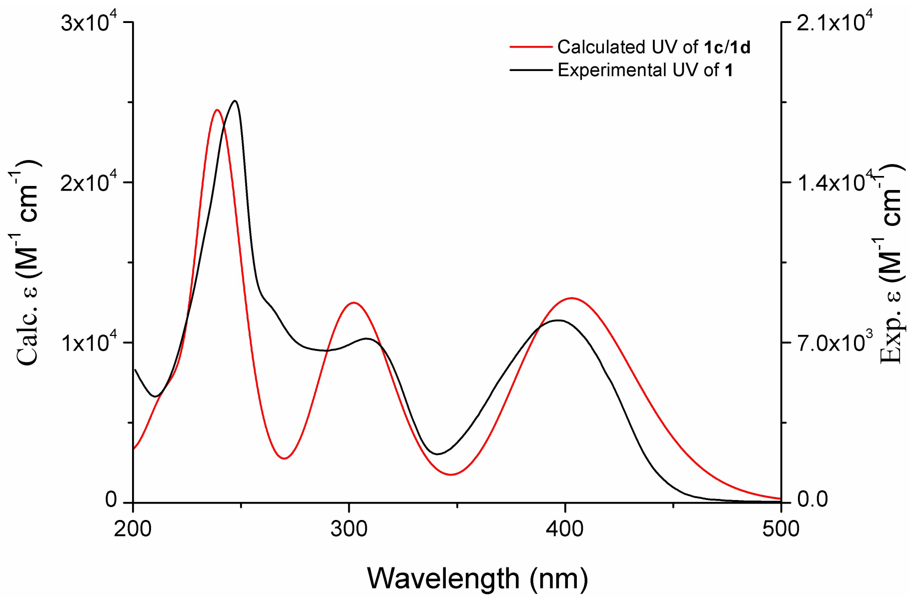

Figure S5. The experiment UV spectrum of **1** and the calculated UV spectrum of **1c**/**1d**.

X-ray crystallography analysis. Using the vapor method, yellow plate crystals of compound **1** were obtained from the system of MeOH-H_2O. A crystal (0.26 mm × 0.38 mm × 0.64 mm) was separated and mounted on a glass fiber which data was collected by Rigaku MicroMax 002+ CCD detector equipped with a graphite monochromator and *CuKa* radiation (λ = 1.54178 Å at 295K). Crystal data as follows: $C_{15}H_{12}O_8$, M = 320.26, space group orthorhombic, $P2_1$; unit cell dimensions, a = 10.760 (2) Å, b = 13.502 (15) Å, and c = 10.990 (13) Å; V = 1502 (4) Å^3, Z = 2, D_{calcd} = 1.536 mg/m^3, F (000) 724. The 11232 measurements yielded 4897 independent reflections after equivalent data were averaged and Lorentz and polarization corrections were applied. Using the SHELXS-97 program the structure was solved by direct methods and expanded using difference Fourier techniques, and refined by the program SHELXL-97 and full-matrix least-squares calculations. The final refinement gave R_1 = 0.0568, wR_2 = 0.1377, S = 1.006. The crystallographic data have been deposited at The Cambridge Crystallographic Data Centre (deposition number CCDC 1407795). The data is available free of charge via www.ccdc.cam.ac.uk/products/csd/ request.

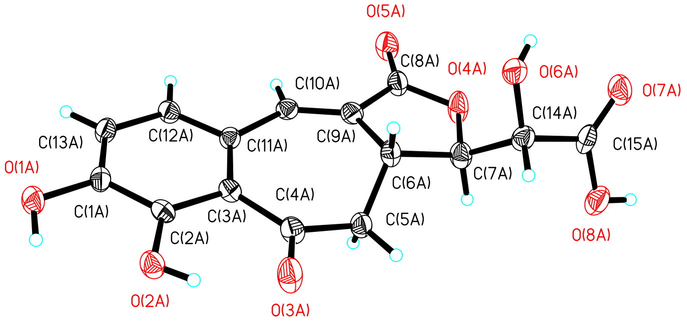

Figure S6. X-ray crystal structure of **1**.

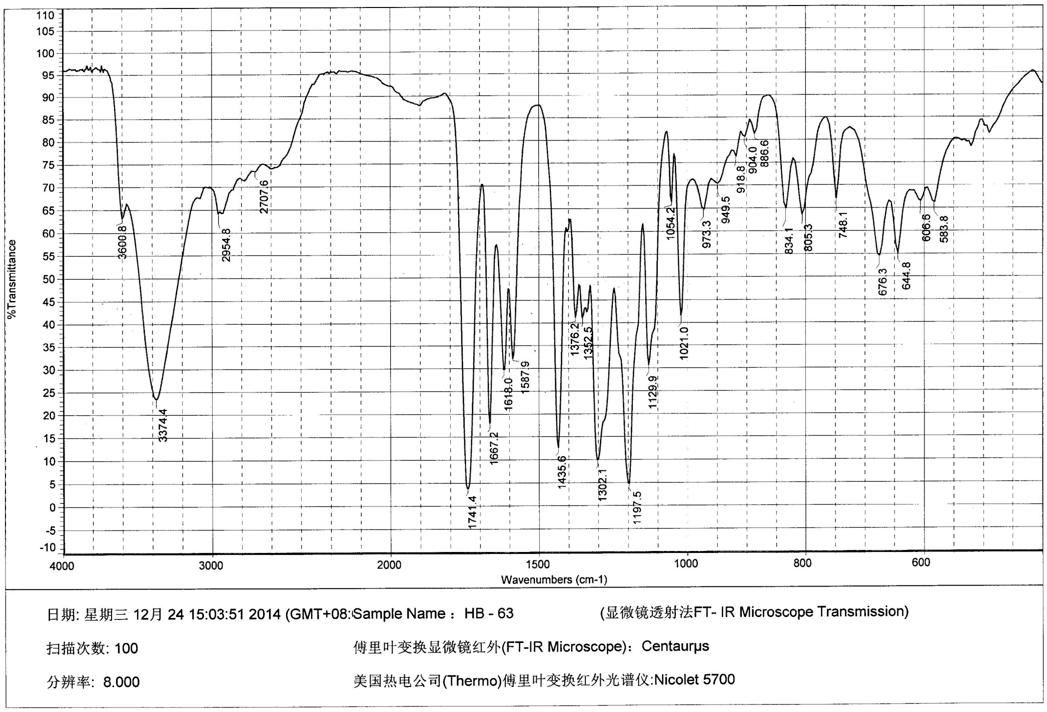

Figure S7.IR spectrum of ligubenzocycloheptanone A (**1**).

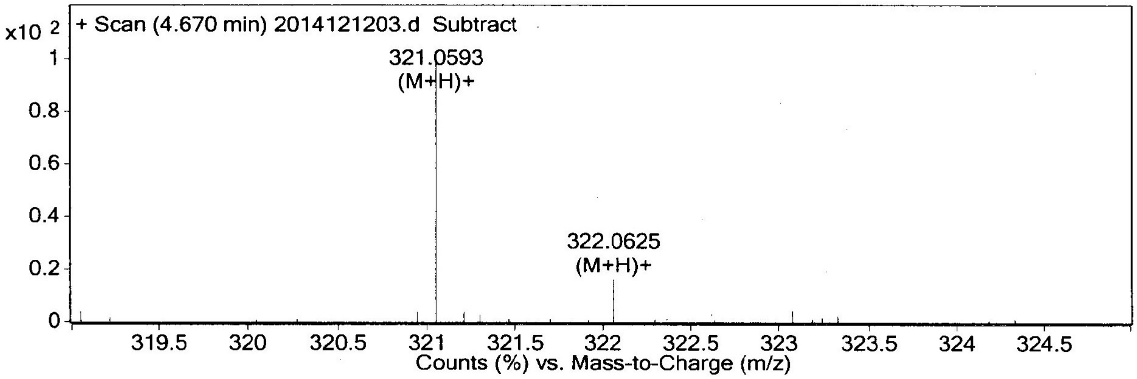

Figure S8. HRESIMS spectrum of ligubenzocycloheptanone A (**1**).

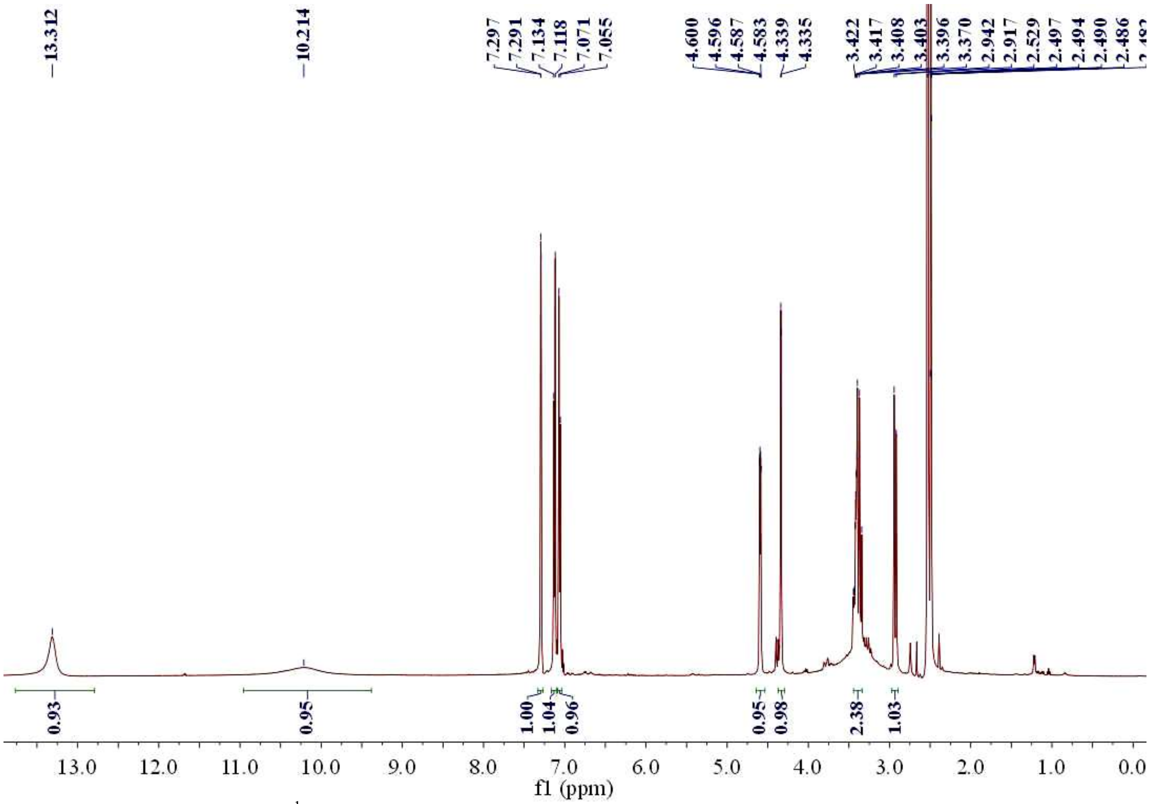

Figure S9. ^{1}H NMR spectrum of ligubenzocycloheptanone A (**1**) in DMSO-d_6.

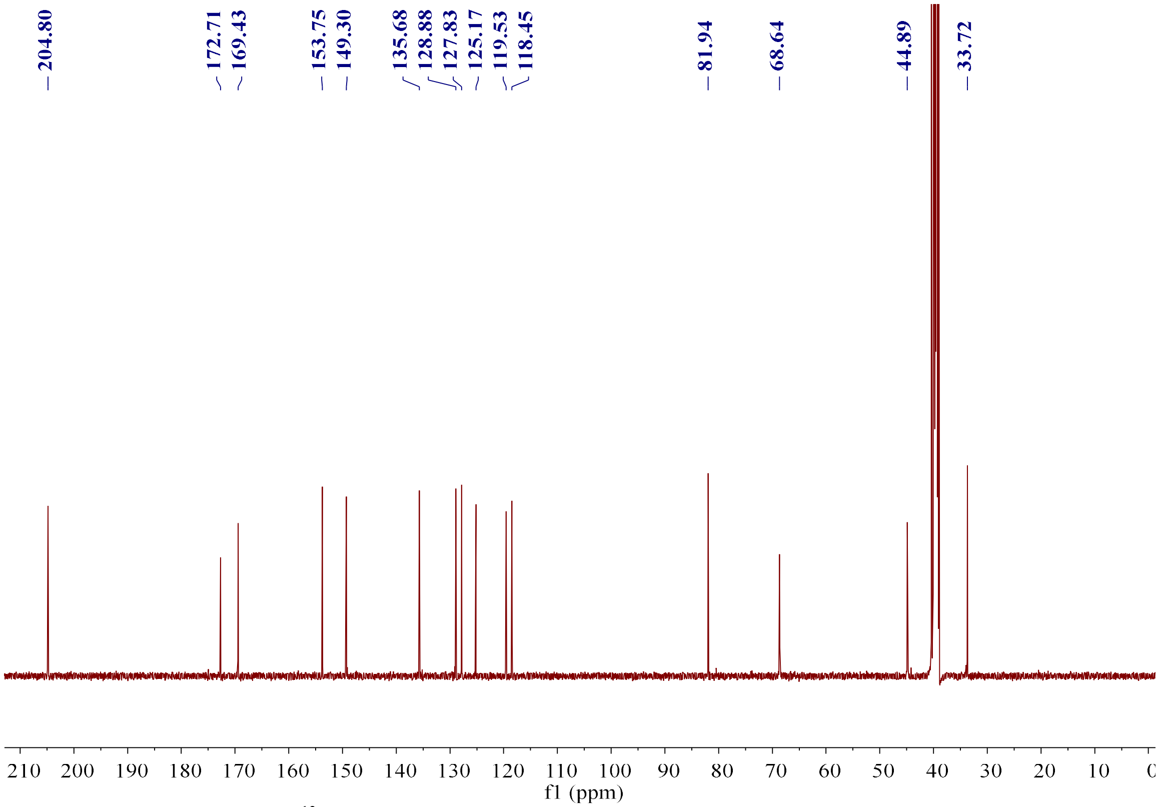

Figure S10. ^{13}C NMR spectrum of ligubenzocycloheptanone A (**1**) in DMSO-d_6.

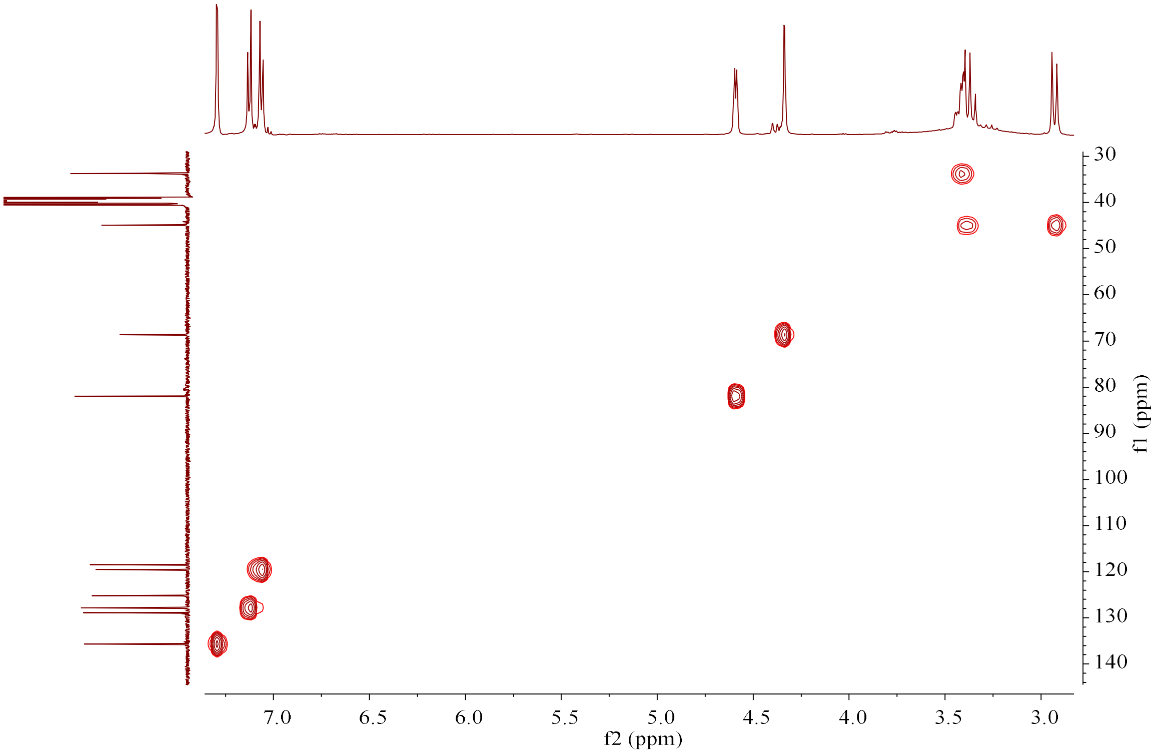

Figure S11. HSQC NMR spectrum of ligubenzocycloheptanone A (**1**) in DMSO-d_6.

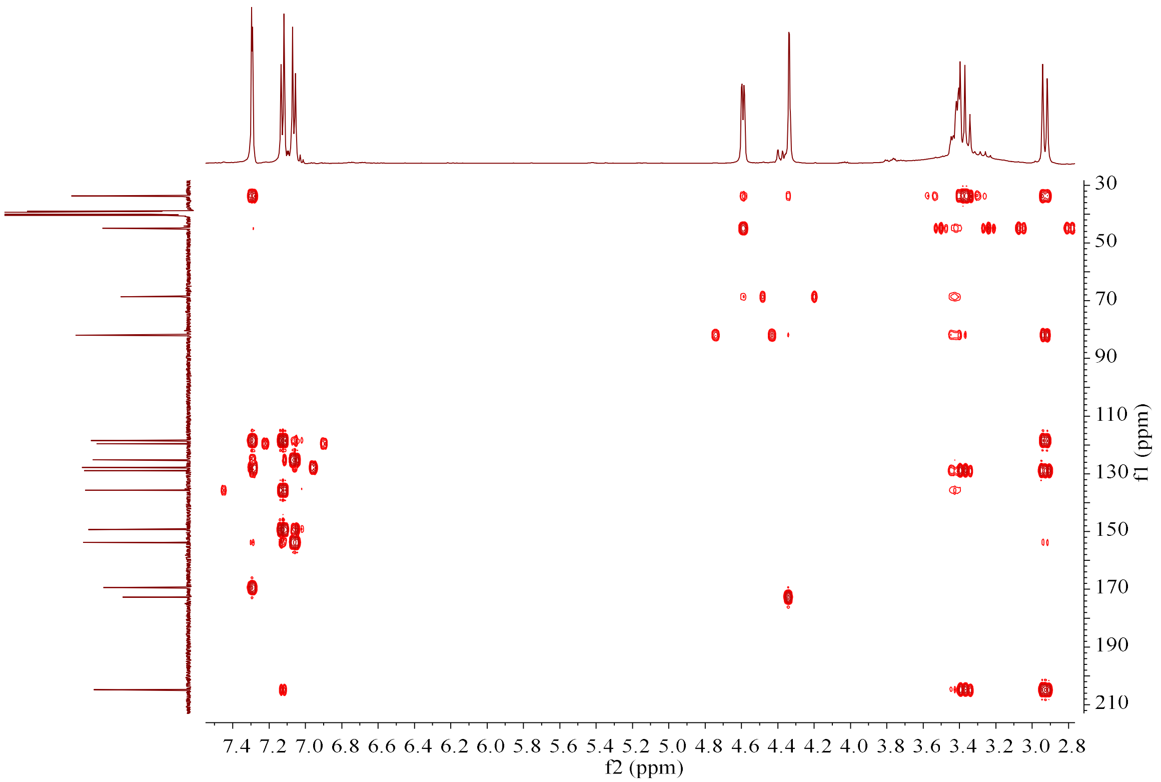

Figure S12. HMBC NMR spectrum of ligubenzocycloheptanone A (**1**) in DMSO-d_6.

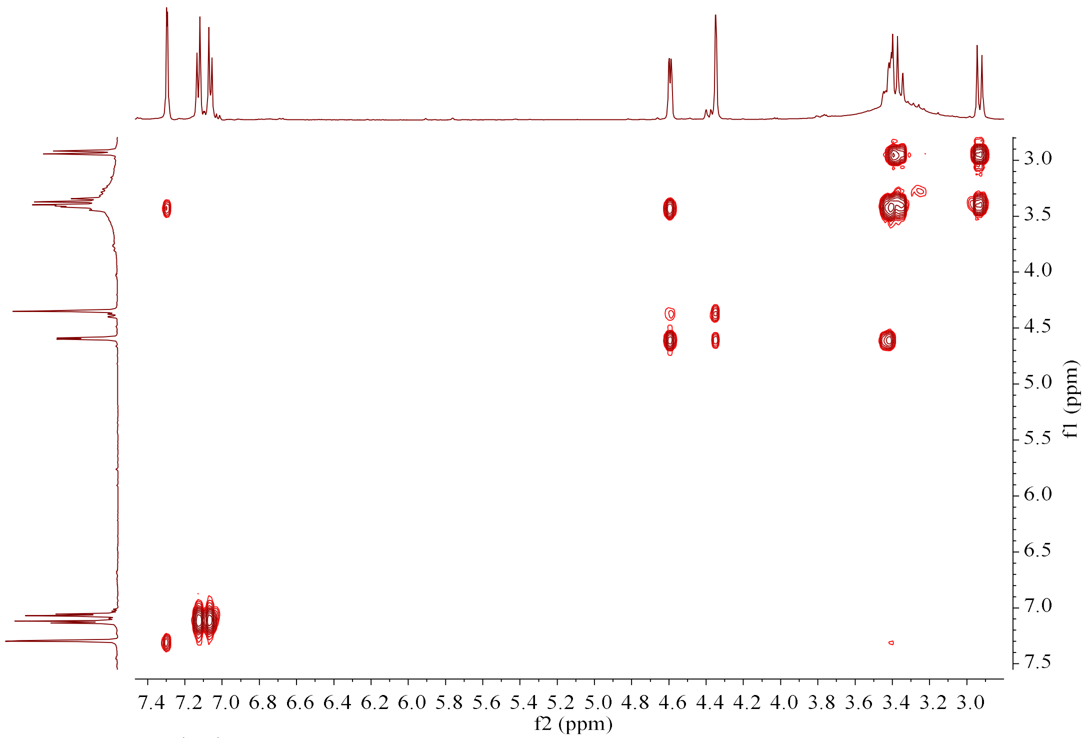

Figure S13. ^{1}H-^{1}H COSY NMR spectrum of ligubenzocycloheptanone A (**1**) in DMSO-d_6.

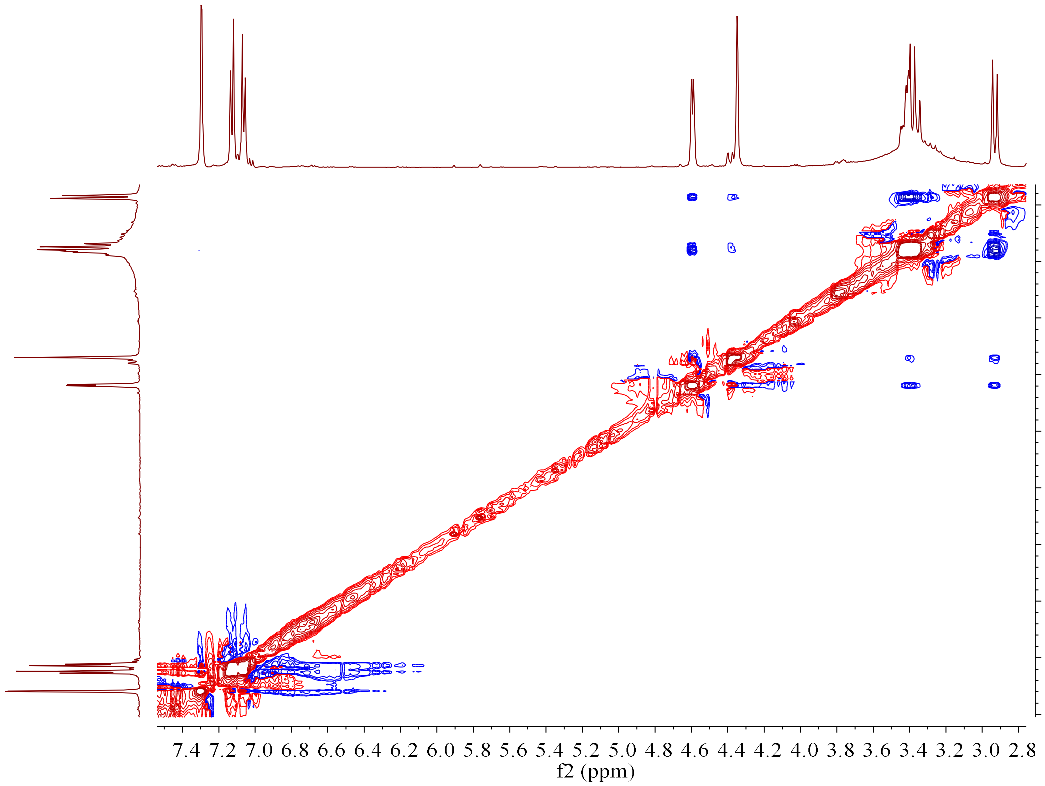

Figure S14. ROESY NMR spectrum of ligubenzocycloheptanone A (**1**) in DMSO-d_6.
